# Gene expression profiles in Finnish twins with multiple sclerosis

**DOI:** 10.1186/1471-2350-7-11

**Published:** 2006-02-27

**Authors:** Silja Särkijärvi, Hanna Kuusisto, Raija Paalavuo, Mari Levula, Nina Airla, Terho Lehtimäki, Jaakko Kaprio, Markku Koskenvuo, Irina Elovaara

**Affiliations:** 1 Neuroimmunology Unit, Department of Neurology, Tampere University Hospital, Teiskontie, 35, 33520 Tampere, Finland; 2 Department of Neurology, Tampere University Hospital, Teiskontie 35, 33520 Tampere, Finland; 3 Laboratory of Atherosclerosis Genetics, Department of Clinical Chemistry, Center for Laboratory Medicine, Tampere University Hospital, and Medical School, University of Tampere, Teiskontie 35, 33520 Tampere, Finland; 4 Department of Public Health, P.O. Box 41, University of Helsinki, FIN-00014 Helsinki, Finland; 5 Department of Mental Health and Alcohol Research, National Public Health Institute, Mannerheimintie 166, FIN-00300 Helsinki, Finland

## Abstract

**Background:**

Since genetic alterations influencing susceptibility to multiple sclerosis (MS), the most common autoimmune demyelinating disease of the central nervous system (CNS), are as yet poorly understood, the purpose of this study was to identify genes responsible for MS by studying monozygotic (MZ) twin pairs discordant for MS.

**Methods:**

In order to identify genes involved in MS development, the gene expression profiles in blood mononuclear cells obtained from eight MZ twin pairs discordant for MS were analyzed by cDNA microarray technology detecting the expression of 8 300 genes. The twins were collected from the Finnish Twin Cohort Study and both affected subjects and their healthy siblings underwent neurological evaluation and cerebral and spinal magnetic resonance imaging. Gene expressions were confirmed by relative quantitative reverse transcription PCR.

**Results:**

It appeared that 25 genes were at least two-fold up-regulated and 15 genes down-regulated in 25% (2/8) of twins with MS when compared to their healthy siblings. Moreover, 6/25 genes were up-regulated in 40% of MS twins and one gene, interferon alpha-inducible protein (clone IFI-6-16) (G1P3), in 50% of them. The six most constantly expressed genes are (1) G1P3, (2) POU domain, class 3, transcription factor 1, (3) myxovirus resistance 2, (4) lysosomal-associated multispanning membrane protein-5, (5) hemoglobin alpha 2 and (6) hemoglobin beta.

**Conclusion:**

Over two-fold up-regulation of these six genes in almost half of MZ twins with MS suggests their role in MS pathogenesis. Studies using MZ MS twins obtained from genetically homogeneous population offer a unique opportunity to explore the genetic nature of MS.

## Background

Multiple sclerosis (MS) is the most common autoimmune demyelinating disease of the central nervous system (CNS) characterised by inflammatory lesions scattered throughout the brain tissue. According to recent data the prevalence of MS in Finland is the highest in the world being 100/100 000, although the figures vary in different regions of the country [1]. The reasons for the increase in the disease prevalence are not known, although viral infections and other environmental factors have been suggested [1].

Family and twin studies have shown that the concordance rate of MS for monozygotic (MZ) twins is about 30% and 2 – 5% for dizygotic (DZ) twins and siblings [2-4]. In a Finnish twin cohort of 15 815 pairs, the concordance rate of MS was 29% in MZ twins and the concordance rate was 0% in DZ twins [5].

It is well known that genetic factors regulate susceptibility to MS, but of these factors only HLA-DR2 has been confirmed to be associated with the disorder [6]. In our previous studies several susceptibility genes and their associations have been reported [7-15]. These are the protective effect of HLA-DR1 and HLA-DR53 combination against MS [9], decreased risk of severe MS of IL-10-1082 AG genotype carriers [12] and high chemokine receptor 5 (CCR5) RNA expression in peripheral blood in primary progressive MS [8]. Increased risk of MS in women has been detected with interleukin-1 receptor antagonist (IL-1RA) allele 2 [11], 5G5G genotype of plasminogen activator inhibitor 1 (PAI-1) gene [10] and interaction between estrogen receptor 1 (ESR1) and HLA-DR2 [13]. Other associations with MS in Finnish population are myelin basic protein (MBP) short tandem repeat [15], intercellular adhesion molecule-1 (ICAM-1) AA genotype (Lys^469^/Lys^469^) [14] and preliminary evidence of two distinct MS susceptibility genes, proximal rs3977 and distal D2S1271-associated genes, on 2q33 outside of cytotoxic T-lymphocyte-associated 4 (CTLA4) gene [7]. Taken together, these observations suggest that experimental approach using MZ twin pairs discordant for MS obtained from Finnish genetically relatively homogeneous population may provide a unique opportunity to explore the genetic nature of MS. Interestingly, one study performed on monozygotic twins with MS reported deficient expression of the inhibitory transcription factor Sp3 in mononuclear blood cells [16].

Since genetic factors influencing MS susceptibility and progression are as yet poorly understood, the purpose of this study was to identify genes responsible for MS development by studying MZ twin pairs discordant for MS identified from the Finnish Twin Cohort Study and using cDNA array technology involving the expression profiles of 8 300 known genes.

## Methods

### Study subjects

We studied eight MZ twin pairs discordant for MS obtained from the Finnish Twin Cohort Study. Patients of MS were identified by linkage to the national hospital discharge registry, which covers all hospitalization in Finland since 1972. All the twin pairs, both affected subjects and their healthy twin siblings, underwent neurological evaluation and magnetic resonance imaging (MRI) of the CNS using 1,5 Tesla MRI unit during a clinical remission of the disease. From the MRI protocol, axial 3 dimensional (3D) T2 fast spin echo (FSE), T1 3D axial spoiled gradient echo (SPGR) and FLAIR sequences were used. T2 hyperintense plaques were analyzed from 3D T2 FSE images, T1 hypointense plaques from 3D T1 SPGR images and FLAIR lesions from FLAIR images. All MS patients showed T1- and T2-lesions characteristic to MS, but Gadolinium-enhanced focal lesions were not detected. The diagnosis of MS was based on Poser's criteria and all diagnoses were definite [17]. The affected twins had no other diseases and their twin siblings were all healthy. The mean age of twin pairs was 51.1 ± 9.1 (SD) years. The neurological disability evaluated by the Expanded Disability Status Scale (EDSS) score was 5.1 ± 1.9 (mean ± SD). The clinical characteristics of twin pairs are shown in Table 1. Four out of 8 twins with MS were treated with interferon beta (IFN-β). One of them had received this treatment for 1 year (patient No2, table 1) and the remaining three patients for two to three years. (patients No3, 5 and 6). In addition most of MS patients had symptomatic medication. The peripheral blood samples were collected during a clinical remission of the disease and the analyses were performed blind to disease status.

**Table 1:** Clinical characteristics of monozygotic twin pairs.

Twin pair no	Gender	Age	Duration of MS (years)	Type of MS	EDSS	Immunomodulatory treatment
1	F	54	11	SP	1.5	n.t.
	F	54	Healthy/0	Healthy		
2	F	48	1	RR	4.0	IFN-β-1a
	F	48	Healthy/0	Healthy		
3	F	55	9	SP	7.0	IFN-β-1b
	F	55	Healthy/0	Healthy		
4	M	53	23	SP	6.5	n.t.
	M	53	Healthy/0	Healthy		
5	F	33	5	RR	4.0	IFN-β-1a
	F	33	Healthy/0	Healthy		
6	F	54	3	RR	4.5	IFN-β-1b
	F	54	Healthy/0	Healthy		
7	M	66	11	SP	6.5	n.t.
	M	66	Healthy/0	Healthy		
8	M	46	22	SP	6.5	n.t.
	M	46	Healthy/0	Healthy		

*F *female; *M *male; *n.t*. no treatment; *EDSS *Expanded Disability Status Scale; *IFN*-β beta-interferon

### Isolation of total RNA

Mononuclear cells were separated from peripheral blood (PBMC) in VACUTAINER^® ^CPT™ Cell Preparation Tubes (Becton Dickinson and Company, Franklin Lakes, N.J., USA) and total RNA were isolated by RNeasy^® ^Mini Kit (QIAGEN, Valencia, CA, USA) according to the manufacturer's protocols. The DNA was removed according to BD Atlas™ Plastic Microarrays (BD Biosciences Clontech, Palo Alto, CA, USA) user manual for DNase treatment of total RNA for 10 μg of RNA, with the exception that RNA precipitation was carried out overnight at -20°C. The quality of total RNA was checked by gel electrophoresis and stored at -70°C until used.

### cDNA microarrays

The study was performed using BD Atlas™ Plastic Human 8 K Microarrays (BD Biosciences Clontech, Palo Alto, CA, USA), which contains duplicate DNA fragments from more than 8 300 known human genes (a list of genes is available at ). 5 μg of total RNA were used for microarray analyses and samples from MZ twin pairs were analyzed at the same time. Microarrays were exposed to phosphoimaging screen and scanned by StormScan 840 Phosphoimager (Molecular Dynamics, Sunnyvale, CA, USA) after 5–7 days exposure time at a resolution of 50 μm to ImageQuant software version 5.1 (Molecular Dynamics, Sunnyvale, CA, USA). The comparison was done for all 8 discordant monozygotic twin pairs and all 8300 genes by using widely used cDNA subtraction procedure according to manufacturer's instructions (Clontech, Palo Alto, CA, USA). In brief, in our array, RNA sample obtained from healthy MZ twin (tissue No1) was compared to corresponding RNA obtained from MZ twin with MS (tissue No2) by using cDNA subtraction method.

### Data analysis and normalization

Analysis was performed using the BD AtlasImage™ 2.7 Beta software (BD Biosciences Clontech, Palo Alto, CA, USA) and data was globally normalized by the sum method. Normalized signal intensities were compared to those of healthy siblings. Ratios of gene expression greater than two-fold were considered significant, based on a 99% confidence interval [18,19]. The data was further analyzed and visualized with the GeneSpring software version 5.0 (Silicon Genetics, San Carlos, CA, USA) and the detailed principles of the cluster analysis and dendrograms can be found from the GeneSpring GX animated tutorial from the internet .

### Quantitative reverse transcription polymerase chain reaction (QRT-PCR)

The cDNA microarray data was confirmed for interferon alpha-inducible protein gene (clone IFI-6-16) (G1P3) by relative quantitative real-time RT-PCR with LightCycler instrument (Roche Diagnostics GmbH, Mannheim, Germany). 1 μg of total RNA was converted into cDNA with Random Primer p(dN)_6 _using 1st Strand cDNA Synthesis Kit for RT-PCR (AMV) (Roche Diagnostics Corporation, Indianapolis, IN, USA). The primers and hybridization probes for QRT-PCR were designed and prepared by TIB MolBiol (Berlin, Germany) and the sequences are the following: forward primer: 5'-GAGTGCAGTGGCTATTCACA-3', reverse primer: 5'-GCGCATGCTTGTAATCCTAC-3', probe 5'-end labeled with acceptor dye LC Red 640: 5'-AGCCTCAAGTGATCCTCCTGTCTCA-3' and probe 3'-end labeled with fluorecein: 5'-CATAGTACACTGCAGCCTCCAACTCC-3'.

The PCR was performed in a 20 μl total volume for target gene with 2 μl LightCycler FastStart DNA Master Hybridization Probes (Roche Diagnostics GmbH), 3 mM MgCl_2_, 0.2 μM each probe, 0.5 μM each primer and 2 μl of cDNA. The PCR was performed with a denaturation at 95°C for 10 min, amplified in 40 cycles of denaturation at 95°C for 10 s, annealing at 60°C for 15 s and elongation at 72°C for 12 s. The cooling was performed at 40°C for 30 s.

Glucose-6-phosphate dehydrogenase (G6PDH) was used as a reference gene. The PCR for this was performed by LightCycler – h – G6PDH Housekeeping Gene Set Kit (Roche Diagnostics GmbH) and was done at the same time and under the same PCR conditions as for the target gene. All reactions for target and reference gene were performed in duplicates. Agarose gel electrophoresis was used to verify the PCR products. QRT-PCR results were calculated by LightCycler Relative Quantification Software with efficiency correction (Roche Diagnostics GmbH).

### Statistical analyses

The significantly expressed genes in our array experiments were defined according to the instructions given by the cDNA array manufacturer (Clontech, Palo Alto, CA, USA). cDNA subtraction ratios of gene expression greater than two-fold were considered significant, based on a 99% confidence interval. The differences of at least 2-fold up- or down-regulated genes within MS twin groups were compared with McNemar test and χ-2 test using statistical software STATA version 8.0 (STATA Corporation, TX, USA) A p-value of less than 0.05 was considered significant.

## Results

### Gene expressions in MZ twin pairs discordant for MS

The numbers of significantly up- or down-regulated genes (at least two-fold difference in expression between twin pairs) in the PBMC of MZ pairs discordant for MS detected by cDNA microarray are shown in Table 2. Comparison between twin pairs showed that 305/8 300 genes were at least two-fold up- or down-regulated in at least 1/8 twins with MS. The proportion of up-regulated genes was significantly higher compared to the proportion of down-regulated genes (p = 0.023 for the difference, χ-2 test). Moreover, 38/305 genes were up- or down-regulated in at least one fourth (2/8) of MS twin pairs (Table 2 and 3). Of these 38 genes, 15 were down-regulated (2 to 10 fold) and 25 up-regulated (2 to 39 fold). 6/25 up-regulated genes were expressed in at least 40% (3/8) of MS twin pairs (Table 2), while none of the 15 down-regulated genes were detected with this high frequency (p = 0.01 for the difference of up- and down-regulated genes in χ-2 test). One gene, interferon alpha-inducible protein (clone IFI-6-16) (G1P3), appeared to be up-regulated in 50% (4/8) of the MZ MS twin pairs.

**Table 2:** Number of up- or down-regulated genes in the discordant twins with MS compared to their healthy siblings.

	Number of twin pairs		
	
	1/8*	2/8**	3/8
Up-regulated	194	25	6
Down-regulated	111	15	0
Total no of genes with change	305	38	6

Genes with at least two-fold change are included.

The proportion of up-regulated genes was significantly higher compared to the proportion of down-regulated genes within twin pair groups *p = 0.023; **p = 0.01 in X-2 test.

**Table 3:** The most constantly expressed genes (n = 38) having at least two-fold change, up- (n = 25) or down- (n = 15) regulation, simultaneously in 2 of 8 discordant identical twins with MS when compared to their healthy siblings.

Gene group/symbol	GenBank accession no.	Description	Up (↑) or down (↓) – regulation of genes
*Basic transcription factors*			
POU3F1	NM_002699	POU domain, class 3, transcription factor 1	↑
NKX2-5	NM_004387	Cardiac-specific homeobox	↑
PHOX2A	NM_005169	Aristaless (drosophila) homeobox	↑
*Cell surface antigens*			
MX2	M30818	Myxovirus (influenza) resistance 2	↑
LY6E	NM_002346	Lymphocyte antigen 6 complex, locus E	↑
NKG7	NM_005601	Natural killer cell group 7 sequence	↓
ITGAL	NM_002209	Integrin, alpha L, lymphocyte function-associated antigen 1 (CD11A)	↓
CD4	M12807	T cell surface glycoprotein CD4 antigen (p55)	↓
*Growth factors, cytokines and chemokines*			
PPBP	M54995	Pro-platelet basic protein	↓
SCGF	D86586	Stem cell growth factor	↓
*Intracellular transducers/effectors/modulators*			
VBP1	NM_003372	Von Hippel-Lindau binding protein 1	↑
STK3	NM_006281	Serine/threonine kinase 3 (STE20 homolog, yeast)	↑ and ↓
*Metabolism*			
HBA1	NM_000558	Hemoglobin, alpha 1	↑
HBA2	NM_000517	Hemoglobin, alpha 2	↑
HBB	NM_000518	Hemoglobin, beta	↑
ECH1	NM_001398	Enoyl Coentzyme A hydratase 1, peroxisomal	↑
ARSA	NM_000487	Arylsulfatase A	↑
HPRT1	V00530	Hypoxanthine phosphoribosyltransferase1	↓
GAPD	X01677	Glyceraldehyde-3-phosphate dehydrogenase	↓
*Ribosomal proteins*			
RPS9	U14971	Ribosomal protein S9	↑
RPS12	NM_001016	Ribosomal protein S12	↑
RPS20	NM_001023	Ribosomal protein S20	↑
RPS29	NM_001032	Ribosomal protein S29	↑
RPL3	NM_000967	Ribosomal protein L3	↑
RPL9	NM_000661	Ribosomal protein L9	↑
RPL39	NM_001000	Ribosomal protein L39	↑
LAMR1	U43901	Laminin receptor 1	↑ and ↓
RPS5	NM_001009	Ribosomal protein S5	↓
RPL27A	NM_000990	Ribosomal protein L27a	↓
RPL30	NM_000989	Ribosomal protein L30	↓
RPL38	NM_000999	Ribosomal protein L38	↓
*Others*			
G1P3	X02492	Interferon-induced protein (6–16, IFI6-16)	↑
LAPTM5	NM_006762	Lysosomal-associated multispanning membrane protein-5	↑
AMH	NM_000479	Anti-Mullerian hormone	↑
GPR6	NM_005284	G protein-coupled receptor 6	↑
BNIP3L	NM_004331	BCL2/adenovirus E1B 19 kD-interacting protein 3-like	↑
ACTB	X00351	Actin, beta	↓

**Figure 1:**
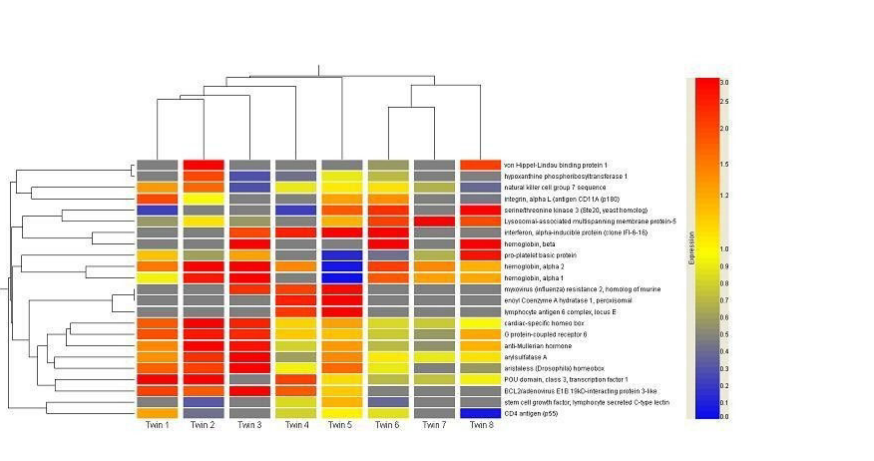
Hierarchical clustering showing relative gene expressions comparing patients with MS to their healthy siblings. Genes with at least two-fold up- or down-regulation in 25% of MS twins are presented. Housekeeping genes and ribosomal protein genes are not included. The colorbar on the right shows the color representation of gene expressions. Grey color indicates the lack of expression in cDNA microarray and twin represents twin pair. The column dendrogram on the left shows the similarity between the expression of different genes and column dendrogram above the similarity in the gene expression profiles of twins. The data was visualized with the GeneSpring software version 5.0 (Silicon Genetics, San Carlos, CA, USA) and the detailed principles of the cluster analysis and dendrograms can be found from the GeneSpring GX animated tutorial from the internet . See also text for the interpretation of the figure.

The hierarchical relationship among ratios of gene expression of 23 most significantly expressed genes between healthy sibling and otherwise identical sibling with MS were compared with cluster analysis. These results are displayed as a dendrogram in Figure 1. As shown by the figure the twin pair samples are roughly divided into two main branches, left branch including twin pairs 1–5 and right branch including twin pairs 6–7. In order to find clinical differences between the branches we characterized these two groups according to the clinical criteria shown in Table 1. From the left branch pairs 1–5 three had SP type (3/5, 60%) and two RR type of MS and from the right branch two of the twins had SP (2/3, 67%) and one RR type of MS (Table 1). Thus these 23 genes were insufficient to separate between relapsing-remitting and secondary progressive MS. From the left branch pairs three (3/5, 60%) of the diseased siblings got IFN-β-1b treatment and from the right branch pair one of the three siblings (1/3, 33%) received this treatment. Also the average EDSS, duration of the MS and mean age of the sibling pairs tended to be lower in the left branch subjects (mean EDSS = 4.6, and mean duration 9.8 years, mean age 48.6 years, respectively) than in the three subjects involved in the right branch (mean EDSS 5.8, mean duration 12.0 years, and mean age 55.3 years, respectively). Furthermore, four of the total five siblings (4/5, 80%) in the left branch were women and in the right branch two of the total tree pairs were men (2/3, 67%).

**Figure 2:**
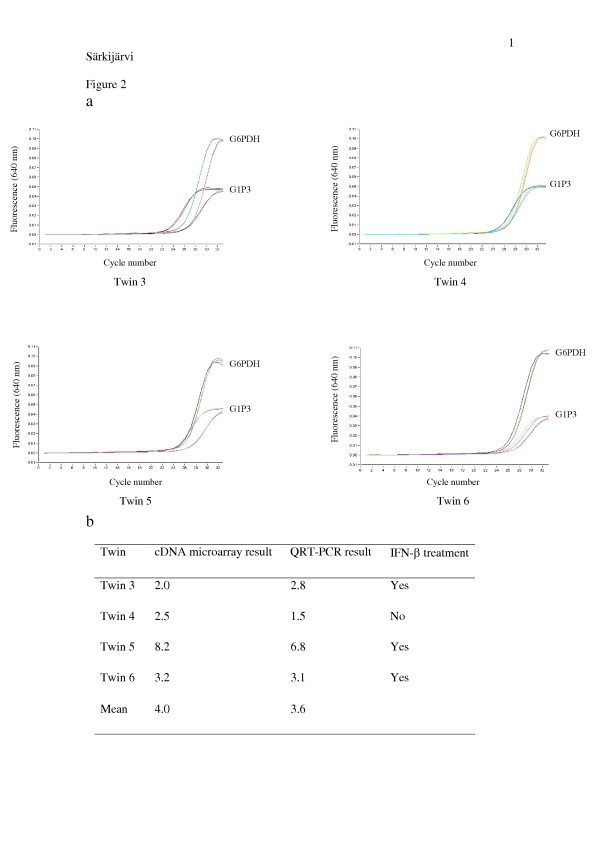
G1P3 gene analyzed by QRT-PCR. Glucose-6-phosphate dehydrogenase (G6PDH) was used as a reference gene in relative quantification. The expression of G1P3 was on average 3.6 times higher in MS twins compared to their healthy siblings, and the results concurred with those obtained from cDNA microarray. Panel **a **shows the real-time QRT-PCR amplifications duplicate in each twin pair (3 – 6) and the table (panel **b**) shows the comparison between the cDNA microarray results and the QRT-PCR results. The y axis indicates fluorescence intensity and x axis PCR cycle numbers. G1P3 gene amplification begins earlier in MS patients than in their healthy siblings, indicating higher gene expression in MS twins.

The six most constantly expressed genes are shown in the Table 4. They are the following: (1) G1P3, (2) POU domain, class 3, transcription factor 1 (POU3F1), (3) myxovirus resistance 2 (MX2), (4) lysosomal-associated multispanning membrane protein-5 (LAPTM5), (5) hemoglobin alpha 2 (HBA2) and (6) hemoglobin beta (HBB). Over two-fold up-regulation of these six genes in 40% of MZ MS twins suggests their role in MS pathogenesis. However, no marked associations between gene expressions and neurological or MRI findings were detected. The expression level of G1P3 confirmed by QRT-PCR appeared to be almost identical with the results obtained by cDNA microarray indicating that our method was working properly (Figure 2).

**Table 4:** The six most constantly expressed genes detected by cDNA microarray.

Gene symbol	GeneBank accession no.	Description	Function	No of twin pairs with up-regulated gene expression
G1P3	X02492	Interferon-induced protein 6–16	Unknown	4
POU3F1	NM_002699	POU domain, class 3, transcription factor 1 (SCIP/Oct-6)	Serves as transcriptional transactivator in the nucleus	3
MX2	M30818	Myxovirus (influenza) resistance 2	Possible antiviral potential	3
LAPTM5	NM_006762	Lysosomal-associated multispanning membrane protein-5	Possible involvement in B cell activation	3
HBA2	NM_000517	Hemoglobin, alpha 2	Oxygen transport	3
HBB	NM_000518	Hemoglobin, beta	Oxygen transport	3

## Discussion

The present study applying modern technique and experimental approach where gene expression profiles of MZ twin pairs discordant for MS were compared to each other revealed differential expression of 305 genes out of 8300 genes studied. Among the differentially expressed genes the proportion of up-regulated genes was significantly higher than that of down-regulated ones (64% vs. 36%). This observation may reflect the balance between immunoactivating and immunoinhibitory factors during the complex inflammatory cascade in MS.

It is noteworthy that up-regulated expression of six genes was found in half of twins with MS. This observation together with the fact that the MS twins were obtained from genetically homogeneous Finnish population suggests the importance of these genes in MS. To the best of our knowledge none of the six genes has previously been reported to be associated with MS. The sample was relatively small, but not unexpected given that MS is relatively rare, as is MZ twinning (about 0.4% of births are MZ twin births), The Finnish Twin Cohort covers virtually all twins alive in 1975 and born before 1958 [20], while the hospital discharge registry identified nearly all MS cases in Finland.

Expression of G1P3 was up-regulated in half of our MS twins. It is known that this gene is transcriptionally induced by IFN-α and -β [21-23], virus infections [24-26] and tumour necrosis factor [27], but its function remains unknown. It is interesting that Ifi-6-16 peptide translated by G1P3 has been identified as an abundant self-peptide induced following measles virus (MV) infection [28], which has previously been associated with MS [29-31]. Since most of our MS twins were treated with IFN-β, the up-regulation of G1P3 in their PBMC can most likely be explained by the IFN-β treatment or some unknown virus infection.

Twins with MS had up-regulated expression of POU3F1. The protein (SCIP/Oct-6) translated by this gene has mostly been studied in the nervous system, where it is associated with Schwann cells in the process of remyelination [32]. In oligodendrocytes, cells producing myelin in the CNS, the SCIP/Oct-6 can stimulate the expression of the papovaviral JC regulatory genes in progressive multifocal leukoencephalopathy (PML) [33], a demyelinating disease of the CNS. However, immune response to the JC virus has not so far been detected in MS.

The MX2 gene was also up-regulated in twins with MS. Mx proteins are induced specifically by IFN-α and -β [34,35] and have antiviral activities [36]. The antiviral potential or other functions of the MxB protein encoded by MX2 gene are not fully understood, while human MxA protein has a wide antiviral spectrum [37] and relatively high levels of its mRNA have been detected after treatment with IFN-β [38]. Since up-regulated MX2 gene was observed in MS twins treated with IFN-β, its up-regulation may be explained by this treatment or alternatively by unknown virus infection.

The up-regulated LAPTM5 gene detected in twins with MS is conserved across evolution but it encodes protein which has no homology to any of the other lysosomal proteins [39].^37 ^LAPTM5 is also known as Clast6 and has been found to be highly expressed in progenitor and precursor B cells [40]. The protein may function during B cell activation or it could have a role in the antigen processing in lysosomes [40], which may be of relevance in MS.

Up-regulated HBA2 and HBB genes detected in MZ MS twins translate proteins that are part of a hemoglobin molecule. Heme units contain iron, which is involved in myelin production by oligodendrocytes and participates in the initiation of oxidative stress-induced injury in the CNS [41]. It is noteworthy that this process plays a role also in the pathogenesis of MS [41].

## Conclusion

Taken together, in this study comparison of gene expression profiles in MZ MS twins to the corresponding profiles of their healthy siblings showed over two-fold up-regulation of six genes in almost half of twins with MS. This observation is of importance taking into account the restricted availability of MZ twins discordant for MS. However, given the sample size, clinical variation among subjects and limitations of the cross-sectional design, our results should be regarded as descriptive and hypothesis generating. To confirm the data MZ pairs discordant for MS need to be studied in other populations. Currently we are in a process of confirming our data with higher number of patients with MS.

## Abbreviations

CCR5 = chemokine receptor 5

CNS = central nervous system

CTLA4 = cytotoxic T-lymphocyte-associated 4 gene

DZ = dizygotic

EDSS = Expanded Disability Status Scale

ESR1 = estrogen receptor 1

G1P3 = Interferon-induced protein

HBA2 = hemoglobin alpha 2

HBB = hemoglobin beta

ICAM-1 = intercellular adhesion molecule-1

IL-1RA = interleukin-1 receptor antagonist

LAPTM5 = Lysosomal-associated multispanning membrane protein-5

MBP = myelin basic protein

MRI = magnetic resonance imaging

MS = multiple sclerosis

MV = measles virus

MX2 = Myxovirus (influenza) resistance 2

MZ = monozygotic

PAI-1 = plasminogen activator inhibitor 1

POU3F1 = POU domain, class 3, transcription factor 1

QRT-PCR = quantitative reverse transcription polymerase chain reaction

## Competing interests

The author(s) declare that they have no competing interests.

## Authors' contributions

SS carried out the experimental work and helped drafting of the manuscript. HK carried out the neurological examination of the patients and participated in drafting of the manuscript. RP, MK and NA participated in the experimental work. TL, JK and MK helped to plan the study and to draft the manuscript. IE was the responsible investigator of the study and participated in planning of the study and drafting of the manuscript.

## Pre-publication history

The pre-publication history for this paper can be accessed here:


